# Interplay Between the Host, the Human Microbiome, and Drug Metabolism

**DOI:** 10.1186/s40246-019-0211-9

**Published:** 2019-06-11

**Authors:** Robert G. Nichols, Jeffrey M. Peters, Andrew D. Patterson

**Affiliations:** Department of Veterinary and Biomedical Science, The Pennsylvania State University, University Park, PA 16802 USA

**Keywords:** Human microbiome, Intestinal microbiome, Oral microbiome, Skin microbiome, Vaginal microbiome, Cytochrome P450, Drug metabolism

## Abstract

The human microbiome is composed of four major areas including intestinal, skin, vaginal, and oral microbiomes, with each area containing unique species and unique functionalities. The human microbiome may be modulated with prebiotics, probiotics, and postbiotics to potentially aid in the treatment of diseases like irritable bowel syndrome, bacterial vaginosis, atopic dermatitis, gingivitis, obesity, or cancer. There is also potential for many of the inhabitants of the human microbiome to directly modulate host gene expression and modulate host detoxifying enzyme activity like cytochrome P450s (CYPs), dehydrogenases, and carboxylesterases. Therefore, the microbiome may be important to consider during drug discovery, risk assessment, and dosing regimens for various diseases given that the human microbiome has been shown to impact host detoxification processes.

## Background

Bacteria, fungi, and other microorganisms together create what is known as the human microbiome, a consortium of various microbial communities located in different niches across the human body [1]. The human microbiome includes the oral, vaginal, skin, and intestinal microbiomes, although the latter two can be further grouped by location to distinguish, for example, the bacteria behind the ear which may be compositionally and functionally different from the bacteria on the back of the hands [2], or to differentiate between the communities of bacteria throughout the gastrointestinal tract [3]. The microbiome composition and function affects the host, whether it enhances food metabolism [4, 5], creates a barrier for defense against dangerous pathogens [6], or provides the host with essential metabolites and vitamins [7]. In addition, the human microbiome composition and function may be associated, although considerable studies are warranted to strengthen these connections, with diseases and disorders ranging from irritable bowel syndrome [8], gingivitis [9], atopic dermatitis [10], outbreaks of bacterial vaginosis [11], oral cancer [12], colon cancer [13], and nonalcoholic fatty liver disease [14]. The discovery of the human microbiome relationship with this wide range of diseases creates unique treatment options and possibilities, and each subsection of the human microbiome can be investigated for potential therapeutic strategies. Exploration of the four major microbiome areas—intestinal, skin, oral, and vaginal—has already begun to support the identification of specific approaches and supplements to potentially modulate major diseases [15].

## Intestinal microbiome

Scientists have known since the late 1950s that the bacteria present in the gastrointestinal tract affect how drugs, toxicants, and host metabolites are metabolized [16]; however, only over the last decade has the intestinal/gut microbiome garnered significant attention [17]. Evaluation of drugs such as levodopa (L-DOPA) [17], lactulose [18], irinotecan [19], and digoxin [20] has identified direct relationships between their efficacy and the intestinal microbiome. Moreover, the advent of high-throughput sequencing has made possible additional investigation of the intestinal microbiome and proven to be a cost-effective analytical technique. This is achieved through decreasing sequencing costs and the ability to sequence hundreds of samples with one sequencing run, which allows for investigation of the potential interactions between the microbiome and host drug response. However, these drugs also illustrate how the intestinal microbiome can hinder the efficacy of their use to treat disease, and only recently have such complications been treated by modulating the intestinal microbiome itself [19]—including the introduction of fecal transplants.

The side effects of extensive antibiotic treatment can include the elimination of the majority of the intestinal microbiome and the creation of a niche for harmful bacteria, such as *Clostridium difficile.* Once acquired, whether through *C. difficile* spores or through native *C. difficile* present in the human intestinal microbiome [21], *C. difficile* grows rapidly and outcompetes the normal intestinal microbiome. This can lead to intestinal complications such as antibiotic-associated diarrhea and colitis, both of which can be severe enough to require hospitalization [22]. *C. difficile* can be treated with vancomycin and metronidazole [22], antibiotics that can provide short-term alleviation of symptoms of the disease. However, *C. difficile* overgrowth can recur due to its spores’ extreme resistance to both vancomycin and metronidazole [23]. Thus, it is not surprising that approximately 20% of patients with diarrhea and colitis treated with vancomycin and metronidazole for *C. difficile* can relapse within 3 weeks post-treatment [23]. This high relapse rate, combined with the increased occurrence of antibiotic-resistant bacterial strains, prompted the development of unconventional treatments including fecal transplants to eliminate reliance on antibiotics.

First proposed in the late 1950s by Dr. Ben Eiseman as a treatment option for pseudomembranous enterocolitis, fecal transplants, later termed fecal microbiota transplants (FMT), have become increasingly used over the past decade [24]. The treatment involves the introduction of a healthy intestinal microbiome into an eligible patient—one with a compromised intestinal microbiome who has relapsed or shown no improvement after antibiotic treatment—via an enema of healthy donor stool [25]. FMT therapy has a 90% success rate on the first attempt [25] and a 98% success rate on the second attempt [26].

Although the US Food and Drug Administration has thus far approved the therapy only for the treatment of resilient *C. difficile* infections, it could be an effective treatment for other intestinal disorders. Research has established a relationship between ulcerative colitis, an irritable bowel disorder involving severe inflammation of the colon and the rectum, and the composition of the intestinal microbiome [27]. A recent clinical trial testing fecal transplants as a potential treatment in patients with diagnosed ulcerative colitis [28] demonstrated a 24% success rate, compared to a 5% success rate for a placebo treatment [28]. Among the major indicators of success was the increased diversity of the intestinal microbiome within the fecal transplant group, which has been associated with a healthier gut [28–30]. Although the success rate appears low, this clinical trial represented only the first attempt of using a fecal transplant as an adequate therapy for ulcerative colitis; additional research will facilitate the evolution of this therapeutic approach and potentially increase its effectiveness.

FMT can also potentially treat metabolic disorders, such as obesity. A 2014 study (4 pairs of twins [one lean and one obese twin], 1 monozygotic set and 3 dizygotic sets) involved the transplant of the obese twin’s intestinal microbiome into a germ-free mouse and the transplant of the lean twin’s intestinal microbiome into the mouse’s germ-free littermate [31]. Although both mice were fed the same diet, the mouse with the obese microbiome became obese, while the mouse with the lean microbiome remained lean [31], demonstrating that the microbiome can potentially promote obesity. However, the question remains whether direct modulation through antibiotics and FMT can attenuate obesity or whether the underlying issues caused by obesity are strong enough to be unaffected by the healthy microbiome.

A recent review evaluated the possibility that obesity can be treated with FMT, as more diversity likely leads to a healthier gut, which in turn leads to a healthier host [29]. However, the mechanism(s) for this effect remain unclear and defining a healthy gut for one individual may be different for another. A recent pilot study examining intestinal microbiome composition before and after a period of fasting [32] showed significant increases in diversity within the same individuals after caloric restriction [32], which has already been associated with a healthy lifestyle [33–35]. Increased intestinal microbiome diversity under caloric restriction could prove beneficial to the host, although additional research must be completed before a definitive conclusion can be drawn. If a strong relationship between microbial diversity and health is established, fecal transplants and extensive microbial modulation could serve as a new and effective treatment for obesity. Since obesity is now a leading risk factor for many cancers [36], FMTs may also be a suitable therapy for treating obesity-dependent cancers [29].

Before FMT becomes a prescribed treatment, there remain a number of unknowns that need to be examined including how the intestinal microbiome modulates gene expression and the activity of proteins/enzymes of the host. Research supports the notion that the bacteria in the intestinal microbiome modulate the expression of genes in the host. This may occur via numerous mechanisms including potential for the gut microbiome to generate metabolites (vitamin B12 and vitamin K) [37], metabolize endogenous metabolites (dehydroxylation of bile acids by *Clostridium spp*.) [38], or modify xenobiotic compounds (bacterial β-glucuronidases used to metabolize xenobiotics) [39]. Interestingly, the downregulation of many host detoxifying enzymes was observed in germ-free mice compared to conventional mice in a study looking at 249 xenobiotic-processing genes in mice [40]. Genes encoding xenobiotic-metabolizing enzymes from several regions of the intestine (duodenum, jejunum, and ileum) and the liver were examined in conventional mice and germ-free mice [40]. Generally, there was different expression across all categories of genes encoding xenobiotic-metabolizing enzymes, supporting the hypothesis that gut microbes influence host xenobiotic-processing genes [40]. Additionally, many cytochrome P450s (CYPs) were decreased in germ-free mice compared to conventional mice, but mRNA levels of transporters (Peptide Transporter/solute carrier 15a1[*Pept1/Slc15a1*], organic cation/carnitine transporter 1/ solute carrier 22a [*Octn/Slc22a*], and multidrug resistance-associated protein 2/ATP-binding cassette [*Mrp2/Abcc2*]) and many phase two enzymes (UDP glucuronosyltransferase 1a1 [*Ugt1a1*], sulfotransferase 2b1 [*Sult2b1*], and Glutathione S-Transferase m7 [*Gstm7*]) were increased in germ-free mice [40]. Interestingly, this differential expression was not specific to the intestinal tissue, but was also seen in the liver [40]. It should be noted that the gene expression changes seen in the aforementioned study, while significant, were smaller than the variation of gene expression typically seen between humans. However, if the gut microbiome does modulate human detoxifying enzyme activity as much as mice, modulation of xenobiotic metabolizing capacity by altered gene expression in the host due to FMT is one potential side effect that needs to be further evaluated. Additionally, over the counter supplements like probiotics need to be investigated for their potential to alter gene expression of the host.

The effects of probiotics on conventional and germ-free mice on the expression of drug-metabolizing enzymes have been recently examined [41]. This study found that many baseline activities of the detoxifying enzymes (CYP4A, CYP3A, CYP1A2, glutathione S-transferases, monooxygenases, carboxyesterases, aldo-keto reductases, and sulfotransferases) were significantly different in the germ-free mice compared to conventional mice [41]. Additionally, supplementation of a probiotic (VSL#3) that contained eight strains of bacteria from the genera *Bifidobacterium*, *Lactobacillus*, and *Streptococcus* modulated the mRNA and protein expression levels of many detoxifying enzymes [41]. Alcohol dehydrogenase 1 (*Adh1*), carboxylesterase 2A (*Ces2*a), and Cyp4v3 were all upregulated after VSL3 treatment, but Cyp3a44, Cyp3a11, glutathione S-transferase mu1, and UDP glucuronosyltransferase family 1 (*Ugt1a*) all decreased after VSL3 treatment [41]. However, it should be noted that dosing with probiotics may be complicated by the fact that mice are coprophagic which may alter the transient colonization of probiotics [42]. However, the results may still be relevant due to the unrestricted nature of how individuals consume probiotics on a near-daily basis. One of the major remaining questions is whether permanent host bacterial modulation is achieved with a single dose of probiotics, or if continued use of probiotics is needed to maintain the changes in host gene expression. This is compounded by the results of a recent study showing that most probiotics only colonize individuals that are deficient in the supplemented strain [42]. Additionally, probiotics were shown to delay recolonization of the human gut microbiome after antibiotic treatment, but had no effect on the mouse microbiome [43]. The different probiotic effects seen between mice and humans is another issue when investigating the effects of probiotics. Furthermore, if one dose of probiotics induces long-term bacterial changes, the baseline host expression of detoxifying enzymes may also change. On the other hand, if continued doses of probiotics are needed to maintain this change, then host detoxifying enzyme expression may be in flux and alterations in drug efficacy, potency, or mechanism of action may occur. Regardless of the answer, drug companies, risk assessment groups, and governmental agencies should consider the common use of probiotics when determining risk factors, doses, and exposure levels of new drugs and toxicants.

Because diet [44], environment [4], and genetics [45] can affect the bacteria present in the gastrointestinal tract, one may hypothesize every individual possesses a unique microbiome composition; as a result, a treatment that works for one human might not work for others [46]. Moreover, all current treatments are focused only on the bacteria residing in the intestinal microbiome, but fungi and viruses make up a very important and often overlooked component of the intestinal microbiome. Future treatments must take all components of the intestinal microbiome into consideration when using it to treat disease—an option made possible by the advent of personalized medicine, which takes a patient’s metabolomic profile, genetic profile, daily activity profile, and microbiome composition into consideration when diagnosing disease and prescribing treatment [47]. Such an approach could make possible specific treatment plans to modulate the intestinal microbiome in a unique way for each individual. However, more studies are required before the impact of the intestinal microbiome for effective disease treatment can be fully understood.

## Skin microbiome

The skin is populated by bacteria, including niches of bacteria specific to certain areas like *Propionibacteriaceae* dominating the microbiome found on the skin on the human back or *Staphylococcaceae* that dominates the heel of the foot and the back of the knee [48]. Commensal bacteria are permanent residents of the skin microbiome. Additionally, there are transient species based on the individual’s current environment [48]. Like the gut, the major phyla of the skin include, *Proteobacteria*, *Bacteroidetes*, *Firmicutes*, and *Actinobacteria*; however, unique to the skin are the major genera *Corynebacteria* (phyla *Actinobacteria*), *Propionibacteria* (phyla *Actinobacteria*), and *Staphylococci* (phyla *Firmicutes*) [2]. The skin microbiome is also made up of fungi like *Malassezia*, which resides mainly on the scalp of healthy individuals [48]. The condition of the skin determines which species reside there—for example, *Staphylococcus* prefers moist areas like the fold of the elbow [48]. The areas behind the ear and on the side of the nose are considered sebaceous and mainly consist of *Propionibacteria* [48]. Finally, skin microbiomes associated with drier sites include the buttocks, arms, and legs, which host mainly *Corynebacterium*, *Enhydrobacter*, and *Micrococcus* species [48]. A complete breakdown of the different bacterial populations in the skin has been previously reviewed [48].

Like the intestinal microbiome, dysbiosis of the skin microbiome can cause significant complications. A common skin infection occurs when *Staphylococcus aureus*, normally a commensal bacterium, overtakes an area and leads to a resistant bacterial infection [2]. This dysbiosis extends to the fungal community of the skin microbiome and scalps with larger numbers of fungal species *Malassezia restricta* that have a greater occurrence of dandruff [49]. In addition, *Propionibacterium acnes*, which protects against normal *S. aureus* and methicillin-resistant *S. aureus* (MRSA) infections [50], can overgrow and lead to outbreaks of acne. Factors contributing to dysbiosis of the skin microbiome include general lifestyle, hygiene, nutrition, age, and even anxiety [48]. Cosmetics [51], chemotherapy [52], and medications used to treat skin disorders can also affect the skin microbiome and cause dysbiosis.

Recent research has reported that purposeful modulation of the skin microbiome may be an effective treatment for some skin disorders. Atopic dermatitis, a common skin disorder that results in patches of itchy, irritated skin, is characterized by an overpopulation of *S. aureus* [53]. In addition, low bacterial diversity of the entire skin microbiome and high populations of fungal species not belonging to *Malassezia* are associated with the prevalence of atopic dermatitis [54]. Moreover, a study reported that babies with very low levels of *Staphylococcus spp.* may be predisposed for atopic dermatitis [55]. This group also reported that delivery method had no effect on the development of this condition [55], which is supported by an additional study showing no associations between birth method and predisposition for atopic dermatitis [56]. Most treatments for atopic dermatitis involve the use of moisturizers to prevent the skin from cracking, but some groups are beginning to supplement that treatment with probiotics. A double-blind study supplemented a moisturizer with the *Proteobacteria* species *Vitreoscilla filiformis* [57] and found it reduced its score on the SCORAD (Scoring Atopic Dermatitis) index—a clinical tool used to determine the severity of atopic dermatitis, with higher scores indicating greater severity—from 31 to 15 in less than 30 days [57]. According to another study, supplementation with coagulase-negative *Staphylococcus spp.* could be used to treat atopic dermatitis [58]. The use of these strains to selectively eliminate *S. aureus* and treat atopic dermatitis is safer than antibiotics [58], which kill a large portion of the skin microbiome and increase vulnerability to opportunistic infections like MRSA. Using supplemented strains to outcompete the *S. aureus* will repopulate with safe, beneficial strains and effectively treat the condition [58]. This group is also attempting to supplement the patient’s own commensal skin bacteria with the moisturizer to help treat atopic dermatitis, although the results are pending [57]. If successful, the use of individual commensal bacteria strains to treat atopic dermatitis will bring science one step closer to truly personalized medicine, for which the human microbiome is an essential element.

Research has reported a correlation between levels of *S. aureus* present on the skin and severity of cutaneous T cell lymphoma [59]. Additionally, baths with low levels of sodium hypochlorite reduce the amount of *S. aureus* on the cutaneous T cell lymphoma patients’ skin [59]. The chronic inflammation caused by *S. aureus* could promote neoplasia in the areas where the bacterial invasions occur [60]. Ultraviolet (UV) radiation causes multiple types of skin cancer, including basal cell carcinomas, squamous cell carcinomas, and malignant melanomas, and one group is attempting to use the skin microbiome as an early risk-detection system for the development of skin cancer from UV radiation [61]. The investigators focused on the production of porphyrin by *P. acnes*, which decreased with increasing levels of UV radiation [61]. Levels of porphyrin can be observed with rapid noninvasive imaging techniques, yielding rapid observations which could be used to determine the severity of UV radiation exposure and guide the necessary treatment [61]. A separate study reported that oral supplementation with the probiotic *Lactobacillus rhamnosus* had a photoprotective effect on the host, delaying skin tumor formation by 4 weeks in a mouse model—the equivalent of 2 years in humans [62].

Like the gut microbiome, the skin microbiome can affect the expression of host gene expression. Again, this is a major factor that is not accounted for in many risk assessment or drug discovery studies, but 16S rRNA amplicon sequencing, metagenomics, and metatranscriptomics may provide additional endpoints to evaluate the impact of the microbiome on host gene expression of the skin. A recent study compared the skin transcriptome of a germ-free mouse and a conventionally raised mouse to examine differential expression of all genes present [63]. Genes associated with RNA processing, metabolism, and transport were reported to be upregulated in conventionally raised mice, but genes associated with inflammation and the immune response were downregulated in conventionally raised mice as compared to the germ-free mice [63]. This study also suggests that the skin and the gut microbiome can cooperate to regulate innate immune function, but it was not known which has a more predominant effect [63]. Upon in-depth analysis of the differential expressed genes, genes that were downregulated in germ-free mice were part of the epidermal differentiation complex [63]. Issues in the epidermal differentiation complex have been reported to cause skin-related disorders like atopic dermatitis and psoriasis [64]. The relationship between the skin microbiome, epidermal differentiation complex, and atopic dermatitis may explain why individuals with lower levels of *Staphylococcus spp.* are more likely to develop atopic dermatitis [55]. The lower levels of *Staphylococcus spp.* could be leading to atopic dermatitis in two different ways: the first being that the absence of *Staphylococcus* spp. is directly causing eczema, and the second is that *Staphylococcus* spp. could be important in the upregulation of the epidermal differentiation complex. Therefore, a decrease in *Staphylococcus* spp. may support a downregulation of the epidermal differentiation complex.

Relationships between the skin microbiome and host gene expression are still being investigated. Additionally, studies are ongoing to probe the use of probiotic creams as treatment and protection for a variety of skin infections. It is known that early exposure to certain species, such as *S. aureus*, can have protective effects for skin disorders like atopic dermatitis [55]. The fungal-bacteria relationship is also an important factor in the treatment for skin disorders such as dandruff, in which increased fungus-to-bacteria levels cause the scalp to become dry and itchy. Overall, the easy access of the skin microbiome for analysis makes it an attractive therapeutic target, but additional research is required to understand the consequences of modulating this population of bacteria.

## Vaginal microbiome

The vaginal microbiome and microenvironment are modulated by pH levels, with different species dominating at varying pH. Vaginal pH is typically between 3 and 4.5, resulting in an acidic environment that helps prevent the invasion of pathogenic species [65]. *Lactobacillus spp.* accounts for almost 70% of the bacteria present in the vaginal microbiome, resulting in a lower pH [65]. Studies report the greater the diversity of the vaginal microbiome, the higher the pH, resulting in vaginal disorders like bacterial vaginosis (BV) and increased risk for the transmission of sexually transmitted diseases [65, 66]. Unlike in the gut microbiome, increased diversity of the vaginal microbiome is a detriment to the host, because *Lactobacillus spp.* are critical to maintain a healthy pH. An increased risk of premature birth has been associated with vaginal disorders during the first trimester of pregnancy, as well as with a lack of *Lactobacillus spp.* within the vaginal microbiome [67]. Unfortunately, the vaginal microbiome remains understudied. As with the other microbiomes, modulating the vaginal microbiome may be an important treatment option when treating various diseases and disorders.

The most common vaginal disorder, bacterial vaginosis (BV), results primarily from the bacteria *Gardnerella vaginitis*, which thrives in a pH environment between 4.5 and 6 [11]. When an increase of diversity in the vaginal microbiota results in a higher pH and an outbreak of *G. vaginitis* [65], BV results, causing itchiness, discomfort, pain, and a foul-smelling discharge [11]. *G. vaginitis* is typically present in most vaginal microbiomes and, like other microbiome bacterial infections, is only a problem if it becomes overabundant. *G. vaginitis* produces vaginolysin (a cytolysin that lyses cells to release nutrients for the bacteria), secretes counter defenses to the host immunity, can be taken up by the host epithelium in times of stress, and creates a biofilm that can inhibit the effectiveness of antibiotics [11, 68]. This makes the bacterial infection difficult to treat with metronidazole, the standard antibiotic for BV treatment [11]. In addition, women who are menstruating regularly have an increased risk for *G. vaginitis*-associated BV, because each menses increases the pH of the vaginal microbiome and reduces the amount of *Lactobacillus spp.* present. Researchers have proposed employing the intrauterine device (IUD)—which prevents regular menses and the associated pH reduction—as a technique for preventing *G. vaginitis*-associated BV [69]. Because the biofilm associated with *G. vaginitis* is one reason BV recurrence rates are so high, another potential treatment could involve biofilm disrupters combined with antibiotics [68]. BV prevalence is also associated with the affected women’s male partners, so simultaneous treatment of both partners could be necessary to effectively treat BV [68]. Prebiotics (supplements that help certain bacteria grow, like fiber) and probiotics, such as lactobacillus supplements, have been used to treat BV with and without antibiotics, but the results have shown no effect [70]—possibly due to colonization issues and a focus on the production of hydrogen peroxide rather than acetic acid [68, 70]. Some groups have explored postbiotics—bacterial metabolites like lactic acid—as a BV therapeutic [70]. Intravaginal lactic acid treatments and intermittent treatment with acetic acid have reportedly been equally or more effective than antibiotic treatment and could be a beneficial therapy for patients affected by persistent BV [71, 72]. Modulation of the vaginal microbiome could prove useful for treating BV in the future, but more research is required.

Like BV, fungal infections are very common and typically occur after antibiotic treatment. These infections are caused by *Candida albicans*, a type of yeast that normally resides in the vaginal microbiome [73]. When the vaginal microbiome is disturbed or the patient is immunocompromised, *C. albicans* enters a pathogenic state and can cause widespread infections [73]. Current treatments for *C. albicans* infections are quite effective, but pathogenic strains can lay dormant, causing recurrent infections [74]. According to a recent study, use of a probiotic strain of *Saccharomyces cerevisiae* can have therapeutic effects on the murine vaginal microbiome with *C. albicans* infections [75]. Probiotic use is a potential replacement treatment for patients who suffer recurrent infections of *C. albicans* infections, for which *S. cerevisiae* works in conjunction with *L. rhamnosus* to produce an inhospitable environment for pathogenic bacterial strains [75]. Bacterial probiotics can be coupled with current treatment strategies to help prevent recurrence [76], as reported in a recent study that investigated common azole treatments for *C. albicans* infections and attempted to use bacterial probiotics to prevent fungal resistance [76]. Results from this study showed that probiotics enhanced the efficacy of common azole treatments and reduced the amount of azole-resistant fungal strains [76]. Bacterial and yeast probiotics both showed no serious side effects and could be easily used as a supplement in patients with recurring *C. albicans* infections [75, 76].

Due to the lack of research on the vaginal microbiome, no studies have been completed on how the vaginal microbiome can affect host gene expression. However, using what was discovered in how the skin and gut microbiome can affect host gene expression, some extrapolations can be made in existing vaginal microbiome studies. One study examined how cytokine levels can lead the vaginal microbiome into a state of dysbiosis and perpetuate BV infections [77]. Increased protein levels of IL-1β, IL-8, and IL-6 were found to inhibit the growth of commensal *Lactobacillus spp*. and lead to colonization by opportunistic pathogens like *S. aureus* [77]. In the skin microbiome, it was observed that germ-free mice produce less cytokines, importantly IL-1β, and germ-free mice exhibited downregulation of genes present in the epidermal differentiation complex [63]. Dysregulation of the epidermal differentiation complex has been shown to lead to atopic dermatitis, as does a decrease in *Staphylococcus* spp. [55, 64]. Potentially, *Staphylococcus* spp. could be involved in host cytokine production, which explains the increases in cytokine production in women with BV and the presence of *Staphylococcus aureus* biofilms. However, it is not known if this is a host-mediated effect or if the vaginal microbiome-mediated effect. This connection could be explored with a vaginal epithelial transcriptome analysis of germ-free mice compared to conventional mice.

## Oral microbiome

The oral microbiome consists of bacteria, fungi, and other microorganisms. Unique to the oral microbiome, however, are three major environments that exist for bacteria and other members of the oral microbiome to colonize [78]. The first and least colonized is the mucosa, which includes the cheeks, gums, and tongue, and its inhabitants are affected by constant epithelial shedding [78]. A healthy oral mucosa consists of *Streptococcus*, *Rothia*, and *Eubacterium* species, but invasions by *Prevotella*, *Porphyrmonas*, and *Tannerella* can lead to the common oral condition halitosis—commonly referred to as bad breath [78, 79]. The second and most diverse area of the oral microbiome is the saliva, which, despite being constantly secreted and swallowed, houses a relatively stable composition of bacteria, fungi, and other microorganisms within each individual (but is highly variable from one individual to the next) [78]. The salivary niche consists of phyla similar to those of the gut, including *Bacteroidetes*, *Firmicutes*, *Proteobacteria*, and *Actinobacteria* [78]. The final microenvironment of the oral microbiome is the teeth, whose hard and solid nature distinguishes them from all other environments within the human microbiome [78]. This environment is ideal for the formation of biofilms, commonly known as plaque, that consist mainly of members from the *Firmicutes* and *Actinobacteria* phyla [78]. The oral microbiome is directly responsible for a variety of common oral disorders, such as dental caries, gingivitis, and periodontitis [78].

Dental caries—also known as tooth decay—is a common oral disorder mainly caused by the bacterial species *Streptococcus mutans* [78], although *Viellonella* [80], *Bifidobacterium* [81], and *Prevotella* [82] species were also observed in greater abundance in children with multiple dental caries compared to those with none. The bacteria associated with dental caries thrive in the low pH environment that can result from a high-sugar diet [83]. These bacteria can then maintain that environment by producing weak acids that demineralize the teeth and make them vulnerable to infection and the development of dental caries [83]. Even small amounts of sugar (2 kg/year) have been observed to modulate the oral microbiome to a caries-prone state [84, 85].

Prebiotics for oral microbiome modulation have not been studied, despite a clear relationship between diet and oral microbiome composition [86]; instead, most studies have focused on probiotics as a means of oral microbiome modulation. One recent study reported a novel *Streptococcus* species, *Streptococcus A12*, has increased arginine deiminase system activity, which can inhibit *S. mutans* in the oral microbiome [87]*. S. A12* could be used as a protective treatment for children at risk of developing dental caries. A large investigation of oral probiotics conducted in 2015 reported that several *Lactobacillus* species (*L. fermentum*, *L. gasseri*, and *L. crispatus*) could adhere to an artificial material that simulates teeth [88]. As a result, the presence of these *Lactobacillus spp.* could drastically reduce the prevalence of dental caries due to the decreased acid production of a high-sugar environment [88]. These studies represent the first wave of potential bacterial treatments for dental caries, and, in the future, probiotics could be used as a preventive measure in children prone to dental caries.

The use of probiotics to treat gingivitis and periodontal disease also warrants exploration. The disease progression begins with gingivitis, an inflammation of the gingiva, or tissues at the base of the teeth. When the gingiva becomes inflamed, the connective tissue holding it to the tooth is destroyed [89], causing the gingival sulcus—the junction between the gingiva and the tooth [89]—to deepen and creating a periodontal pocket that can harbor bacteria. If left untreated, this pocket can destroy teeth and the surrounding bone [89]. Gingivitis patients have been found to possess increased amounts of *Campylobacter*, *Prevotella*, and *Fusobacterium* and decreased levels of the dominant *Streptococcus spp.* [78]. The transition from gingivitis to periodontal disease involves a major shift of microbial composition in the periodontal pocket that can increase the diversity of this pocket by almost 400 species [90]. Both gingivitis and periodontal disease are dependent on the bacterial composition of the oral microbiome, presenting an opportunity to explore the use of probiotics to decrease risk and treat the diseases. According to one recent study, the use of a probiotic lozenge inoculated with *L. rhamnosus GG* and *Bifidobacterium lactis BB-12* improved the periodontal state of the mouth by decreasing the plaque [91] and gingival [92] indexes without affecting the species of the oral microbiome [93]. A yearlong clinical trial tracked patients using a variation of these lozenges, inoculated instead with *Lactobacillus reuteri*, as a periodontal treatment [94]. After a year, the group using the probiotic lozenge had smaller periodontal pockets, and fewer of the patients required surgery [94], leading the clinicians to conclude that use of the probiotic lozenge was a viable, noninvasive treatment for individuals suffering from chronic periodontal disease [94].

Considering how variable individual oral microbiomes are from one another, how the oral microbiome can affect host gene expression should be an area for concern to researchers involved with risk assessment, drug discovery, and toxicant exposure. However, the only research into how the oral microbiome can modulate host gene expression is with the biofilms that can form on the teeth, particularly biofilms that cause periodontal disease [95]. The area where the teeth connect to the gum is known as the gingival margin, and harmful biofilms can grow above (supragingival) or below (subgingival) this margin. An investigation into the potential of both biofilms reported modulation of host gene expression, particularly via the inflammasome [95]. The supragingival and subgingival both had different effects on the host gene expression, but both biofilms could increase the mRNA expression of cytokines [95]. Of particular note, the subgingival biofilms were reported to actively downregulate *NLRP3* (nucleotide-binding oligomerization domain-like receptor P3), one of the major inflammasomes in the body, which can increase survival for the biofilm [95]. How biofilms modulate host gene expression was also examined using gingival cell lines exposed to a single species of bacteria, multiple species, and dead bacteria/biofilms [96]. Each type of biofilm and type of bacteria used produced different host responses. The more mature the biofilm was, the stronger the host pro-inflammatory response [96]. Unfortunately, there are limited studies examining other microbes in the mouth and their effects on host gene expression, despite other research showing that microbes found in the gut and on the skin can affect host gene expression.

## Conclusions

As noted above, the intestinal microbiome can markedly influence the effectiveness of certain drugs. First reported in the 1970s, Dr. Barry Goldin discovered that when supplementing germ-free rats with L-DOPA, the major metabolites of L-DOPA, *m*-tyramine, and *m-*hydroxyphenylacetic acid (*m*-HPAA) were not detected in the urine [17]. Dr. Goldin concluded that the intestinal microbiome was responsible for the metabolism of L-DOPA, and without the intestinal microbiome, L-DOPA has an increased half-life and efficacy [17]. Certain drugs like lactulose require metabolism by the intestinal microbiome, resulting in their therapeutic metabolites, acetic and lactic acid [18]. Other drugs like irinotecan are reactivated by the intestinal microbiome via the β-glucuronidase enzyme present in many intestinal bacterial species, resulting in serious side effects in the host such as stage 4 diarrhea and gastrointestinal damage which typically requires hospitalization [19]. The above examples are from studies of the intestinal microbiome. Unfortunately, few drug metabolism studies have been done using the oral, vaginal, and skin microbiome. Additionally, a recent review investigated the presence of CYPs in different taxonomic families, from humans to bacteria [97]. This review reports that bacteria have a diverse collection of CYPs that differ drastically from bacterial species to species, and when compared to the 57 different CYPs in humans, bacteria have approximately 3000 different CYPs [97]. The disproportionate number of bacterial CYPs compared to human CYPs prompts a question as to whether any bacterial species have CYP-like proteins that match human CYP activity. In other words, do bacteria have CYP orthologues that could modulate host drug metabolism?

Modulation of the human microbiome will undoubtedly play a role in the future of precision medicine. The growth of genetic sequencing capabilities, rapid increase in computing power, and growing focus on the individual all drive scientific research to enhance our understanding of the human microbiome. While many probiotic, antibiotic, prebiotic, and even postbiotics therapies are currently in use to treat and prevent myriad diseases and disorders throughout the human body, they require more research before they can be validated as effective options. Research into the human microbiome will usher in a new era of personalized medicine, in which customized treatments will be employed with the recognition that each individual’s human microbiome reacts uniquely to treatments. Individualized treatment will depend on a combination of the complete human microbiome composition, the individual genetic makeup, environmental factors, and dietary elements to create personalized treatment plans.

## Data Availability

Not applicable

## Abbreviations

Adh1: Alcohol dehydrogenase 1
BV: Bacterial vaginosis
Ces2a: Carboxylesterase 2A
CYPs: Cytochrome P450s
ExPASy: Expert Protein Analysis System
FMT: Fecal microbial transplants
GMQE: Global Model Quality Estimation
Gstm7: Glutathione S-Transferase m7
IL: Interleukin
IPA: 3-Indole propionic acid
IUD: Intrauterine device
L-DOPA: Levodopa
LPS: Lipopolysaccharide
m-HPPA: Hydroxyphenylacetic acid
Mrp2/Abcc2: Multidrug resistance-associated protein 2/ATP-binding cassette
MRSA: Methicillin-resistant *S. aureus*
NLRP3: Nucleotide-binding oligomerization domain-like receptor P3
Octn/Slc22a: Organic cation/carnitine transporter 1/solute carrier 22a
Pept/Slc15a1: Peptide Transporter/solute carrier 15a1
QMEAN: Qualitative Model Energy ANalysis
SCORAD: UGT1a Scoring Atopic Dermatitis
Sult2b1: Sulfotransferase 2b1
UDP: Glucuronosyltransferase family 1
Ugt1a1: UDP glucuronosyltransferase 1a1
UV: Ultraviolet
